# Role of CEACAM1, ECM, and Mesenchymal Stem Cells in an Orthotopic Model of Human Breast Cancer

**DOI:** 10.4061/2011/381080

**Published:** 2010-11-07

**Authors:** Sridhar Samineni, Carlotta Glackin, John E. Shively

**Affiliations:** ^1^Irell & Manella City of Hope Graduate School of Biological Sciences, Duarte, CA 91010, USA; ^2^Department of Immunology, Beckman Research Institute of City of Hope, Duarte, CA 91010, USA; ^3^Department of Neurosciences, Beckman Research Institute of City of Hope, Duarte, CA 91010, USA

## Abstract

Carcinoembryonic antigen-related cell adhesion molecule-1 (CEACAM1) is a morphogen in an *in vitro* model for lumen formation and plays a similar role in breast epithelial cells implanted in humanized mammary fat pads in NOD-SCID mice. Although extra cellular matrix alone is sufficient to stimulate lumen formation in CEACAM1 transfected MCF-7 cells grown in 3D culture, there is an additional requirement for stromal or mesenchymal cells (MSCs) for these cells to form xenografts with glandular structures in an orthotopic site. We demonstrate that optimal *in vitro* conditions include both Matrigel and MSCs and that the inclusion of collagen I inhibits xenograft differentiation. Additionally, there is no need to remove the nascent murine mammary gland. The previously observed difference in gland development between the long and short cytoplasmic domain isoforms of CEACAM1 is no longer observed in pregnant NOD/SCID mice suggesting that stimulation of the mammary fat pad by pregnancy critically affects xenograft differentiation.

## 1. Introduction

Mammary gland development is critically dependent on mesenchymal tissue [1, 2]. In the correct context, mammary epithelial cells will develop branched glandular tissue capable of milk production. In order to study human breast cancer in an animal model, it is necessary to implant breast cancer cells in an orthotopic site along with human stromal cells and other components such as ECM. The identification of essential components for proper growth of breast cancer epithelial cells was pioneered by Bissell and coworkers in an *in vitro* model in which mammary epithelial cells are grown in a 3D culture of extracellular matrix supplied by Matrigel [3, 4]. However, this model lacks the contribution of mesenchymal cells (MSCs) that are implicated in both normal mammary gland development and breast cancer [1, 5]. Recently, Kuperwasser et al. [6] developed an *in vivo* model in NOD/SCID mice in which the nascent murine mammary gland was removed and immortalized human breast fibroblasts were introduced to humanize the gland prior to addition of human mammary epithelial cells. Other components of the model include the use of radiation-killed breast fibroblasts, Matrigel, and collagen I. In our own studies, we have found that the human mammary epithelial cell line MCF7 that lacks CEACAM1 fails to form glands with a lumen in the *in vitro* 3D model, while transfection of CEACAM1 into MCF7 cells restores gland-like formation [7]. Similarly, when wild type MCF7 cells are grown in humanized mammary fat pads of NOD/SCID mice, solid tumors are formed, while CEACAM1 transfected MCF7 cells form glands with a lumen [8]. Surprisingly, we found a difference in gland formation between the two major isoforms of CEACAM1, namely, the short (forms no glands) versus the long (forms glands) cytoplasmic domain isoforms. This difference has led us to speculate that the ratio of short to long isoforms in human breast may become altered in breast cancer leading to the altered morphology characteristic of ductal carcinoma in situ (DCIS) [9]. These results have also prompted us to dissect the phosphorylation status of the long and short cytoplasmic domains in further experiments in the humanized mammary fat pad model [8, 10]. During the course of these studies, we began to question the role of each of the components used in the humanization of the murine mammary fat pad and the physiological status of the gland.

In a comprehensive analysis of the model, we have found that removal of the nascent murine mammary gland is not required to obtain well-differentiated glandular xenograft formation for CEACAM1 transfected MCF7 cells. We also show that immortalized human MSCs can be used in place of the immortalized breast fibroblasts and that there is no requirement for either collagen I or radiation-killed breast fibroblasts. However, the inclusion of Matrigel and MSCs appears to be essential while the inclusion of collagen I inhibits xenograft differentiation. Interestingly, the aforementioned short isoform of CEACAM1 (CEACAM1-4S), that failed to form glands in the original model, formed glands in pregnant NOD/SCID mice, indicating that the physiological status of the gland is also important.

The role of MSCs in breast cancer development has been recently explored by the Weinberg group [11] where they demonstrated that MSCs can promote breast cancer metastasis for certain breast cancer cell lines grown subcutaneously in mice. We show here that MSCs, in this case immortalized MSCs, are not necessarily prometastatic as predicted by Karnoub et al. [11], but instead can promote differentiation and glandular development of epithelial cells in the xenograft.

## 2. Materials and Methods

This study was approved by the City of Hope/Beckman Research Institute Institutional Animal Care and Use Committee (IACUC) and Institutional Biosafety Committee (IBC). All animal experiments were conducted in COH/BRI Animal resource center, which is AAALAC accredited and is in compliance with the Animal Welfare Act and the *Guide for the Care and Use of Laboratory Animals.* Human fetal bone marrow collection was approved by Institutional Review Board (IRB).

### 2.1. Cell Cultures

Breast cancer cell lines, MCF-7, MDA-MB-468 (MEM medium with L-glutamine, 10%FBS, 1% Sodium bicarbonate, 1% sodium pyruvate, and 1% antibacterial-anti-mycotic solution), and DU4475 (RPMI-1640 Medium supplemented with L-glutamine, 10% FBS, and 1% antibacterial-anti-mycotic solution), were obtained from American-type culture collection and SUM1315 cellline (F-12 medium supplemented with L-glutamine, 10% FBS, 5 *μ*g/mL insulin, and 10 ng/mL epidermal growth factor, and 1% antibacterial-anti-mycotic solution) was a gift from Dr. Michael Rosenblatt, Tufts University, School of Medicine. *Selection of stable cell lines:* transfection and selection of MCF-7 cells with CEACAM1-4S or vector were previously described [12].

#### 2.1.1. Stromal Cell Lines

Immortalized breast fibroblasts (RMF/EG) were a gift from Dr. Kuperwasser, Tufts University (DMEM medium supplemented with L-glutamine, 10% calf serum, and 1% antibacterial and antimycotic solution) and MSC cell line (alpha-MEM medium supplemented with L-glutamine, 15% FBS, and 1% antibacterial-anti-mycotic solution). To generate the MSC cell line, bone marrow cells (MSCs) were obtained from human fetal long bone and cells were filtered through nylon mesh and grown in 60 mm culture dishes with Modified Eagle medium alpha (MEM-*α*) supplemented with 15% fetal bovine serum and 25 mg/mL gentamicin (feeding medium). Primary MSCs were immortalized with human telomerase (hTERT) in the pBABE-neo-hTERTretroviral vector (addgene Inc). Polyclonal populations of immortalized MSCs (hTert MSC) were selected through serial passages in media containing neomycin. The hTERT MSCs were characterized by FACS analysis for cell surface markers and were positive for CD44, CD90, CD105, CD146, CD166, ALP, and STRO-1. These cells can differentiate in vitro into the adipocytes, chondrocytes, myocytes, and osteoblasts depending on differentiation media (data not shown).

### 2.2. In Vivo Studies

The breast cancer cells (0.2 million cells) and/or stromal cells (0.2 or 0.05 million cells) in extracellular matrix (50 *μ*L-Matrigel or Matrigel plus type 1 collagen or PBS) were injected into orthotopic sites (humanized orthotopic mammary fat pad site or mouse mammary stroma (no clearing of fat pad and humanization)) of NOD/SCID mice. The total number of animals in each group was 8 and each animal has two xenografts, for a total of 16 xenografts. The results shown are representative of the results.

#### 2.2.1. Removal and Humanization of Mammary Fat Pad

The humanized orthotopic mammary fat pad mouse model developed by Kuperwasser et al. [6] was used except for the substitution of single cell suspension for mammospheres, and MSCs instead of immortalized human fibroblasts. Briefly, three-to four-week-old NOD/SCID mice were anesthetized with isoflurane and placed on dorsal recumbency. The abdominal and inguinal area was shaved and cleaned with betadine and 70% alcohol. After disinfection of the skin, small midline incision (1 cm) close the fourth nipple by small scissors was made. Forth inguinal mammary fat pads were carefully excised using iris scissors (clearance of mouse mammary fat pad) and the incision was closed with super glue. Animals received analgesics (buprenorphine, *s.c*) and antibiotics (Trimethoprim Sulfa in water) following surgical procedures. Two weeks later, 2 × 10^5^ unirradiated MSC cells plus 2 × 10^5^ irradiated MSCs (50 × L in saline) were injected into the cleared mammary fat pad (humanization of fat pad). Human breast cancer cells (2 × 10^5^ cells) plus 2 × 10^5^ MSCs in 50 *μ*L of collagen type I and matrigel mixture (3 : 1) were injected into the humanized area. Then, mice would be observed for tumor growth for 4–12 weeks and mice were euthanized and the tumors were collected and examined for morphology and expression of CEACAM1.

### 2.3. Human Breast Cancer Tissue Implantation

Human breast tumor tissue for these experiments was procured in compliance with NIH regulations and reviewed and approved by Institutional Review Board (IRB#05091), City of Hope National Medical Center. Deindentified tissue was subjected to either enzymatic digestion (collagenase and hyalurondinase, Stem cell technologies) to isolate epithelial cell organoids according to the manufacturer's recommendations or cut into the small pieces (0.1 cm) to implant into the orthotopic site of the NOD/SCID mice. In case of organoids, 20–25 human breast epithelial organoids were mixed with 2 × 10^5^ hTert MSC in 50 *μ*L of Matrigel and injected into the orthotopic site of NOD/SCID mice (four animals per specimen). For breast cancer tissue implantation, three- to four-week-old NOD/SCID mice were anesthetized, surgically prepared and cared for as indicated in Section 2. Briefly, small midline incision close to fourth nipple was made and small piece (0.1 cm) of breast tumor tissue was immersed in Matrigel containing hTert MSC (1 × 10^6^/mL) and placed underneath the mammary fat pad (orthotopic site) and skin incision was closed with superglue.

### 2.4. Histology

Xenografts were collected and fixed in 10% phosphate buffered formalin and subject to hematoxylin and xyline (H and E) staining.

### 2.5. Statistical Analysis

Statistical significances between the groups were analyzed by One-way ANOVA (Tukey's multiple comparison test).

## 3. Results

### 3.1. Comparison of Xenograft Growth in an Orthotopic Site with or without Nascent Glands

In the model described by Kuperwasser et al. [6], the nascent mammary gland was removed from the fat pad of NOD/SCID mice prior to implantation of first immortalized stromal cells followed by implantation of stromal cells plus epithelial mammospheres. Since many groups have implanted tumors at the mammary fat pads without the prior removal of the nascent mammary gland [13, 14], we first investigated the requirement for nascent gland removal. In our model system, we utilized wild type or CEACAM1-4S transfected MCF7 (MCF7/CEACAM1) cells implanted as a single cell suspension rather than as mammospheres because we felt that the preformation of mammospheres may already resemble gland formation. Thus, we were interested in asking if, in the correct environment, single cells could organize themselves into gland-like structures without the prerequisite of mammosphere formation. Except for this one change, we followed the Kuperwasser model in one set of mice which had the nascent murine mammary gland removed, inoculation with viable plus irradiated stromal cells (hTert MSC) followed by inoculation with MCF7 or MCF7/CEACAM1 cells plus the mixture of stromal cells (hTert MSC) plus collagen I and Matrigel (3 : 1 mixture) (see Table 1 for experimental details). In the other set of animals, the nascent murine mammary gland was not removed. Thus, these mice directly received MCF7 or MCF7/CEACAM1 cells plus the mixture of stromal cells plus collagen I and Matrigel (3 : 1).

**Figure 1:**
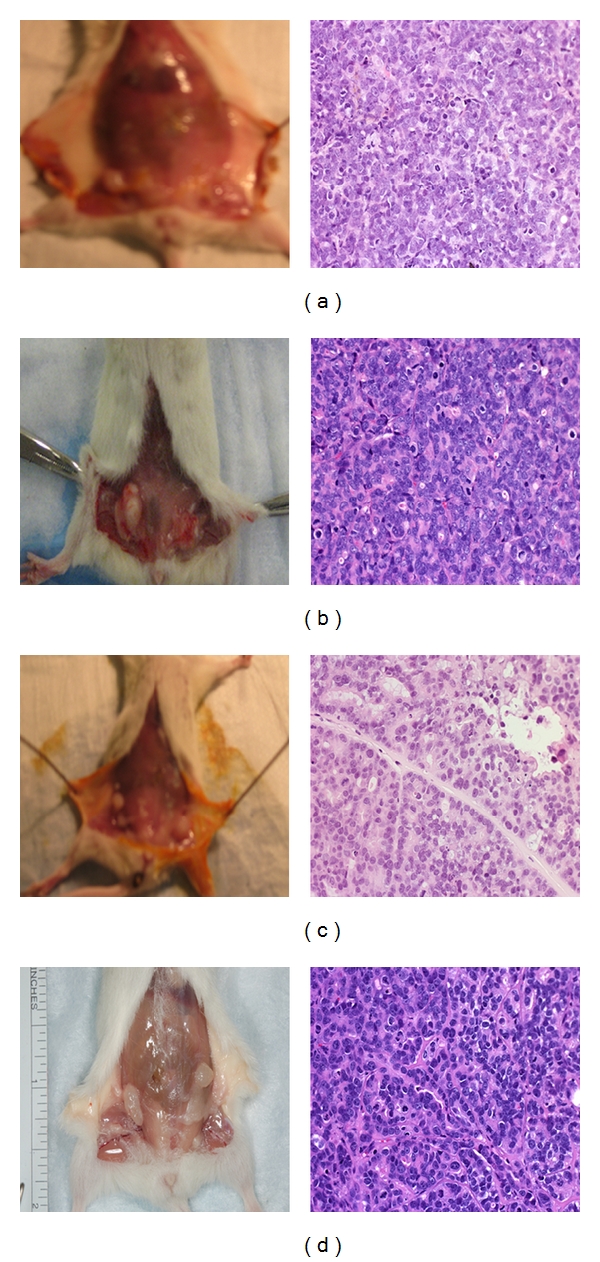
Comparison of human mammary xenograft formation by MCF-7 and MCF7/CEACAM1 in either humanized mammary fat pad or nascent mammary fat pad in NOD/SCID mice. Gross pictures (left panels) of human xenografts and corresponding H and E stained sections (right panels) of MCF7 xenografts (a) in humanized mammary fat pads or (b) MCF7 xenografts in nascent mouse mammary fat pads) or (c) MCF7/CEACAM1 xenografts in humanized mammary fat pads or (d) MCF7/CEACAM1 xenografts in nascent mouse mammary fat pads. Magnification 200x.

Human xenografts from the humanized mammary fat pad model were palpable around 10–12 weeks for both MCF7 and MCF7/CEACAM1 xenografts. When the mice were euthanized and the skin dissected, we observed the close association of the xenograft and the nascent mammary fat pad. However, the xenograft tissue was distinct from the murine mammary fat pad, indicating that the two tissues were not comingled. Histological examination of MCF7 xenografts revealed solid tumors (poorly differentiated carcinoma) without any evidence of higher organization or differentiation and with very little incorporation of extracellular matrix (Figure 1(a)). In contrast, MCF7/CEACAM1 xenografts were organized and differentiated into a striated epithelium-like structure with abundant extra cellular matrix between the cells, resembling moderately differentiated carcinoma (Figure 1(b)). These results are identical to those previously published by us in this model [8] with the difference that hTert/MSC cell line was used in place of immortalized human breast fibroblasts (RMF/EG). In the case of xenografts in which the nascent mammary gland was not removed, the xenografts grew more rapidly and were palpable within 8 weeks (Figures 1(c) and 1(d)). MCF7 and MCF7/CEACAM1 xenografts were of similar size and had the same histological features as xenografts from the humanized mammary fat pad model. To confirm that xenografts were originated from human cells and not comingled with the mouse mammary tissue, we performed immunohistochemistry on the histological section with human cytokeratin antibody and human CEACAM1 antibody as previously described in our paper [8], and all xenografts stained positive for human cytokeratin while only MCF7/CEACAM1 xenografts stained positive for human CECAM1 (data not shown). These results indicate that the MCF7 cells (with or without CEACAM1) grow equally well in the orthotopic sites of both humanized mammary fat pad and nascent mouse mammary pad of NOD/SCID mice and have identical histological features. Thus, at least in this model, there is no need to remove the nascent mammary gland. In the humanized mammary fat pad model, not only is surgery required for removal of the nascent mammary gland followed by two weeks of recovery, humanization with human stromal cells for two weeks is also required followed by 10–12 weeks for growth of xenografts. Thus, a total of 16–18 weeks is required per animal per experiment. In contrast, xenograft growth at the orthotopic site of the unresected mouse mammary fat pad requires only 6–8 weeks for collection of xenografts. 

### 3.2. Xenografts Using other Breast Cell Lines and Human Breast Cancer Tissue

**Figure 2:**
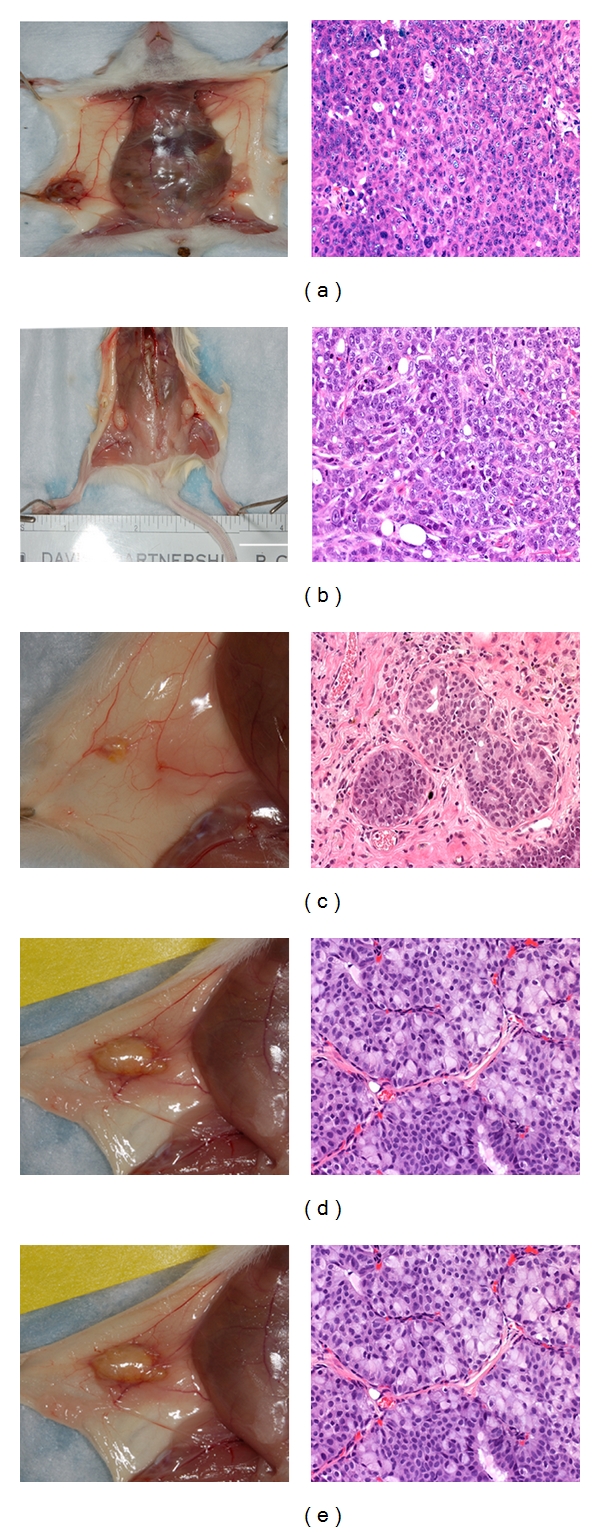
Growth and formation of xenografts by commonly used breast cancer cell lines or human breast tumor tissue in nascent mammary fat pad of NOD/SCID mice. Gross pictures of human xenografts (left panels) and corresponding H AND E stained sections of xenografts (right panels) from breast cancer cell lines or human breast tissue implantation (a) SUM1315, (b) MDA-MB-468, (c) DU4475 (Magnification 200x), (d) human breast epithelial organoids (Magnification 400x), and (e) human breast tumor slices (Magnification 200x).

To examine whether breast cancer cell lines other than MCF-7 would grow in unresected mammary fat pads, we established xenografts using MDA-MB-468 [15], SUM1315 [16], and DU4475 [17] breast cell lines. Xenografts were collected at 8 weeks or when outgrowth observed. We found that all three breast cancer cell lines formed palpable tumors within 4–6 weeks, and interestingly, the DU4475 cell line took only 3-4 weeks for tumor outgrowth (>10 mm). Histological examination of these tumors revealed that DU4475 and MDA-MB-468 tumors incorporated extracellular matrix and had some degree of organization (Figures 2(b) and 2(c)). In particular, DU4475 tumor cells were organized into small clusters with a nesty pattern separated by extracellular matrix (Figure 2(c)). Similar to the DU4475 xenografts, MDA-MB-468 xenografts were palpable around 6 weeks with no organization of tumor cells with extracellular matrix. In contrast, xenografts from SUM1315 cells grew as solid tumors (poorly differentiated carcinoma) without incorporation of extracellular matrix and with good vascularization (Figure 2(a)). When human breast xenografts were isolated eight weeks after implantation of either epithelial organoids or small slices of breast tumor tissue at mammary fat pads of NOD/SCID mice, xenograft outgrowths were observed. The xenografts from epithelial organoids were small (1-2 mm), highly vascularized, and consistently produced tumor outgrowths with features of ductal carcinoma in situ (Figure 2(d)). In the case of breast tumor tissue slice implantation, xenografts were larger (4-5 mm) with features of invasive carcinoma (Figure 2(e)). These observations indicated that human breast tissues or human breast cancer cell lines with appropriate stromal environment can be grown in an orthotopic site of NOD/SCID mice.

### 3.3. Effect of ECM and Stromal Cells on Xenograft Growth

**Figure 3:**
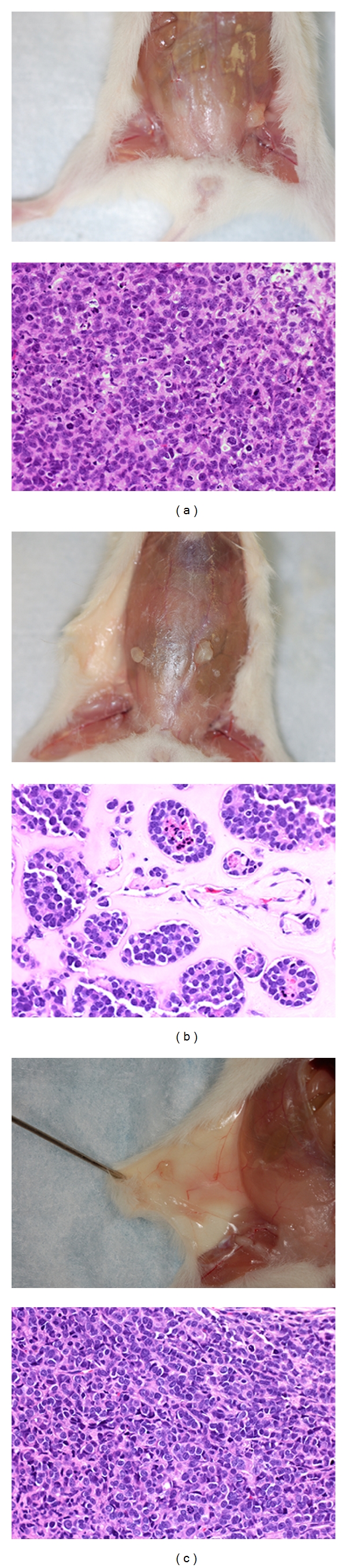
Effect of ECM and stromal cells on growth and differentiation of MCF7/CEACAM1 xenografts. Gross pictures of human xenografts (left panels) and corresponding H AND E stained sections of xenografts (right panels) containing MCF7/CEACAM1 cells in mouse mammary fat pad with (a) hTert/MSC and no ECM, (b) ECM (Matrigel alone) and no stromal cells, and (c) ECM ( Collagen 1 and Matrigel 3 : 1 ratio) and no stromal cells. Magnification 200x.

One of the unexplained features of the humanized mammary fat pad model is the requirement for both Matrigel and collagen I during the engraftment of the breast cancer epithelial cells. It should be noted that the 3D model of mammary morphogenesis developed by Lee et al. [3] contains Matrigel and no collagen I. In fact, collagen I is not a normal component of extracellular matrix (which has collagen IV) and its inclusion in the engraftment protocol is unexpected. In addition, the requirement for both stromal cells and ECM is unexplained; that is, if stromal cells can produce their own ECM, why is there a need for exogenous ECM? To determine if the mixture of Matrigel plus collagen I influences the organization and differentiation of the mammary epithelial cells into glandular structures, MCF7 cells (or MCF7/CEACAM1) with no ECM, Matrigel alone or collagen I plus Matrigel (3 : 1) were inoculated into nascent mammary fat pads along with stromal cells (see Table 2 for experimental details). First, we found that implantation of either cell line with no ECM resulted in low engraftment of xenografts, where only three out of eight animals developed tumors and xenografts that were small (<1-2 mm) compared to xenografts from mice that received ECM (Figure 3(a)). Histological examination of these xenografts revealed that there was no high level of organization or differentiation of MCF7/CEACAM1 cells with very little ECM between the cells, whereas xenografts that included ECM were organized into striated epithelium and had abundant ECM. These results demonstrate that ECM is necessary for engraftment and differentiation of xenografts.

 Next we addressed the issue of stromal cell requirement. MCF7/CEACAM1 xenografts in which ECM included collagen I with no stromal cells engrafted; however, xenografts were small and there was no differentiation or organization of epithelial cells into striated epithelium (Figure 3(c)). Moreover, MCF7/CEACAM1 cells in Matrigel alone (no stromal cells) also engrafted, and although the size of xenografts was small (<1-2 mm), the omission of collagen I from the ECM mixture promoted mammary epithelial cell differentiation into solid acini formation lacking lumen, resembling hyperplastic normal mammary gland (Figure 3(b)). These results indicate that inclusion of stromal cells is necessary for expanded growth of xenografts and that ECM is required for engraftment and initial differentiation of the epithelial cells. In addition, the composition of ECM influences the organization and degree of differentiation of the epithelial cells, but in which the inclusion of collagen I inhibits the further differentiation of epithelial cells into acini with lumen. Thus, stromal cells and ECM are necessary for optimal growth and differentiation of human xenografts. Based on these results, we used Matrigel alone with no collagen I for further experiments.

### 3.4. Effect of Stromal Cell Type on the Model System

**Figure 4:**
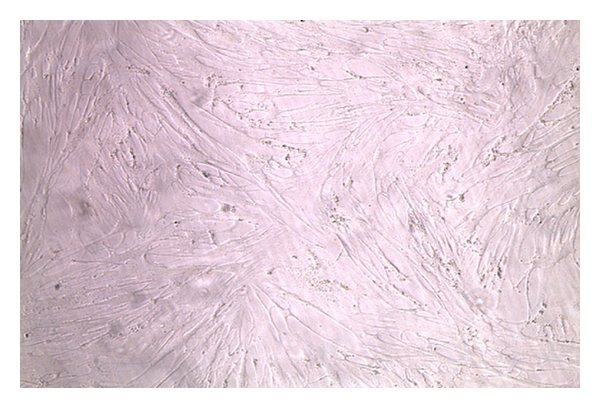
Morphology of hTert/MSC cell line. Phase contrast photograph of hTert/MSC cells grown on plastic.

In the original study of Kuperwasser et al. [6], an immortalized human breast fibroblast cell line (RMF/EG) was used as the source of stromal cells. In later studies, Weinberg and coworkers [11] studied the effect of bone marrow derived mesenchymal stem cells (MSCs) on the growth and metastatic behavior of breast cancer cell lines. Both the requirement for and the source of the stromal cells, including their ultimate effects on the model system, are relatively unexplored. In order to address this issue, we developed a human bone marrow derived MSC cell line (hTert/MSC) and tested its ability to substitute for the RMF/EG cells used in the original model. The morphology of the hTert/MSC cell line was typical of stromal cells (Figure 4(a)). A major impetus for substituting hTert/MSC cells for RMF/EG cells was that they do not required the addition of irradiated stromal cells, a requirement that was unexplained in the model of Kuperwsser et al. [6].

**Figure 5:**
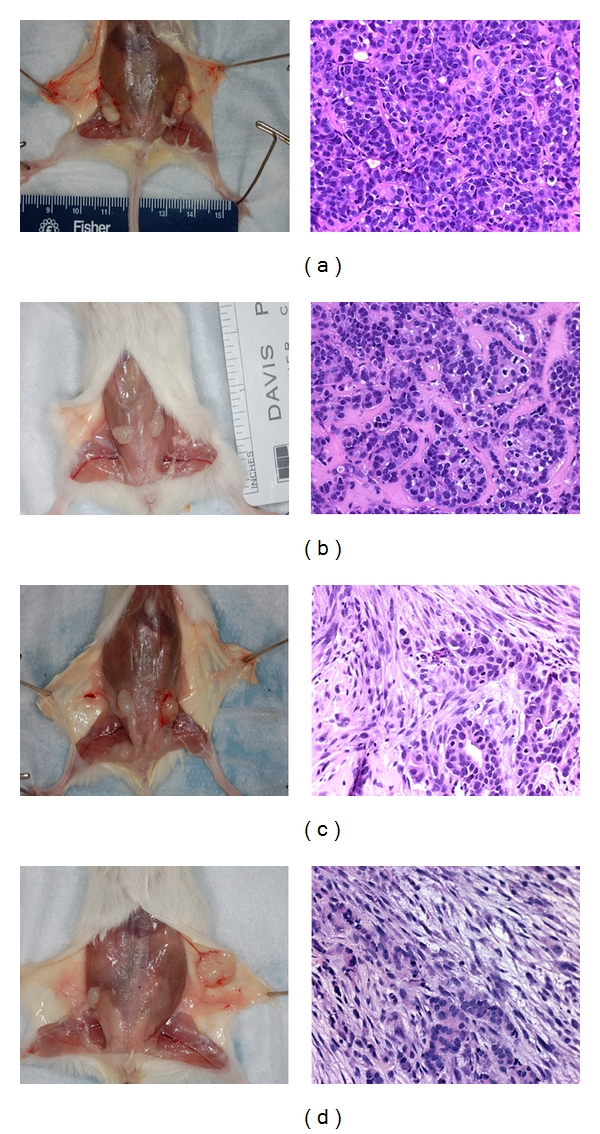
Effect of type and number of stromal cells on the growth and differentiation of MCF7 and MCF7/CEACAM1 xenografts. Gross pictures of human xenografts (left panels) and corresponding H and E stained sections of xenografts (right panels) with hTert/MSC or immortalized breast fibroblasts (RMF/EG) and different ratios of epithelial cells: stromal cells. (a) hTert/MSC : MCF/CEACAM1 ratio 1 : 4 in Matrigel, (b) hTert/MSC : MCF7/CEACAM1 ratio 1 : 1 in Matrigel, (c) RMF/EG along with MCF7/CEACAM1 in Matrigel (1 : 1 ratio), and (d) RMF/EG along with MCF7 in Matrigel. Magnification 200x.

The hTert/MSCs (hTert/MSC to MCF7/CEACAM1 at a ratio: 1 : 4 or 1 : 1) or immortalized human breast fibroblasts (RMF/EG to MCF-7 or MCF7/CEACAM1 ratio: 1 : 1) in Matrigel (no collagen I) were implanted into mammary fat pads (no prior humanization) (see Table 4 for experimental conditions). hTert MSCs (both ratios) promoted growth of the epithelial cells in the xenografts; however, xenografts from 1 : 1 hTert MSC: MCF7/CEACAM1 cell ratio had more extracellular matrix between the glandular structures and had the appearance of ductal hyperplasia (Figures 5(a) and 5(b)). Notably, xenografts with a hTert/MSC:epithelial ratio of 1 : 4 had decreased ECM and differentiation of epithelial cells into ducts filled epithelial cells, suggesting that a ratio of ≥1 should be used. Although human breast fibroblast (RMF/EG) containing xenografts grew faster (palpable within 4 weeks) and larger than the xenografts with hTert/MSCs (10 mm versus 5 mm), histological examination revealed the overgrowth of fibroblasts and the lack of proliferation of epithelial cells, for both MCF7 and MCF7/CEACAM1 (Figures 5(c) and 5(d)). Importantly, implantation of RMF/EG immortalized human breast fibroblasts alone also led to the formation of fibrosarcomas but this did not occur for hTert/MSCs (data not shown).

### 3.5. Effect of Physiological Status of the Mammary Gland on Xenografts

**Figure 6:**
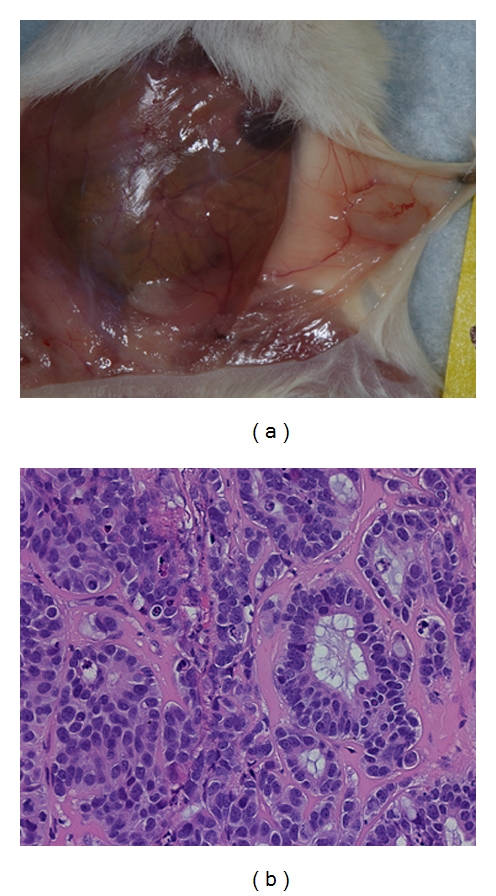
Effect of physiological status of the mammary gland on growth and differentiation of xenografts. MCF7/CEACAM1 cells along with hTert/MSC (1 : 1 ratio) in Matrigel were implanted into the inguinal mammary fat pads of eight-week-old female NOD/SCID mice and immediately mated to induce the pregnancy to change the physiological status of mammary gland. After weaning of pups, (a) xenografts were collected and (b) H and E staining performed on sections from xenografts. Magnification 200x.

Murine mammary glands are maintained in a physiologic nascent state until pregnancy. Once the animal is pregnant the gland rapidly expands in size, increasing the number of ducts and alveolar structures [18]. In humans, the breast is more developed following puberty than in mice, raising the possibility that major functional differences are likely between murine and human postpubescent mammary glands. Since the physiologic status of the gland is expected to have an impact on the growth of implanted xenografts [19, 20], it was important to test our final model in pregnant mice as a surrogate for a postpubescent human mammary gland. To understand the impact of pregnancy on xenografts formation and differentiation, MCF7/CEACAM1 cells along with hTert MSC in matrigel were transplanted into inguinal mammary gland of eight-week-old NOD/SCID mice; mice were mated immediately and then xenografts were collected after the weaning of pups. These xenografts were well vascularized and histological examination revealed formation of well-differentiated glands with a central lumen (Figures 6(a) and 6(b)). These results demonstrate the importance of physiological status of in vivo system on the differentiation of the xenografts.

In summary, we examined the role of stromal cells and ECM in the human xenograft growth and breast cancer epithelial cell differentiation. We found that nascent mammary gland removal and its humanization were not necessary for growth and differentiation of human xenografts, and MSC and ECM were required for the growth and engraftment of xenografts. ECM composition is also important for differentiation and organization of mammary epithelial cells, and presence of Collagen I inhibits differentiation of epithelial cells (summarized in Table 3).

## 4. Discussion

Although considerable progress has been made in the development of an *in vitro* 3D model of human mammary morphogenesis, the 3D model lacks the physiological setting of a live animal that includes features such as the cellular components of the immune system and angiogenesis, or hormonal influences from the endocrine glands, ovary, and the mammary gland itself. The recently developed model of Kuperwasser in which the mammary glands of NOD/SCID mice were “humanized” was a major step forward permitting the growth of human mammospheres in a more physiological setting [6]. However, this model requires complete removal of the nascent mammary gland, takes a long time to establish the “humanized” gland with both radiation-killed and live-immortalized stromal cells, involves both Matrigel and type I collagen, and utilizes mammospheres rather than single-cell suspensions to establish new glands. We have examined each of these features and developed a more streamlined model that greatly speeds the development of xenografts (4–8 weeks versus 16–18 weeks). Other features of our improved model include the retention of the nascent murine mammary gland, obviating the need to wait for the repopulation of the mammary fat pad by human stromal cells. We found that the xenografts grow as solid tumors with no evidence of comingling with the nascent murine mammary gland. When the skin was peeled back at the end of 4–8 weeks of growth, we found that the xenografts adhered to the inner surface, while histological analysis revealed varying degrees of tissue organization depending on the presence or absence of stromal cells and Matrigel. Importantly, while the inclusion of type I collagen in the matrix appeared to inhibit overgrowth of the RMF/EG stromal cells, the use of an MSC cell line obviated the need for collagen I and resulted in xenografts that were comprised primarily of epithelial cells. Nonetheless, both stromal cells and Matrigel are required for proper establishment of xenografts, emphasizing the critical role of ECM in the development of mammary glandular tissue.

MCF7 cells are weakly tumorigenic and do not grow well in subcutaneous transplants unless the mice are treated with estrogen [21]. However, this cell line can be an attractive model for studying breast cancer because reintroduction of genes such as CEACAM1 can cause reversion to a more normal phenotype [7, 8]. For example, the degree of differentiation of the mammary epithelial glands depends on the expression of critical morphogens such as CEACAM1 [8]. MCF cells lacking CEACAM1 always produced solid tumors in either the original “humanized” mammary fat pads of Kuperwasser et al. [6] or in the improved model described here. In contrast, MCF7 cells transfected with CEACAM1 produced either striated or single-layered epithelial sheets, depending on the physiological environment. The dramatic conversion of multilayered epithelial sheets to single-layered mammary glands required pregnancy, emphasizing the role of hormonal status in developing mammary glands. The resulting mammary glands were surrounded by ECM suggesting that ECM production by stromal cells is also dependent on the physiological status of the tissue. In nonpregnant mice, less ECM was produced and the mammary epithelial cells were multilayered, suggesting that a communication program exists between the epithelial and stromal cells that is context dependent. The context probably includes, among other hormones, the production of prolactin by the pituitary in the pregnant mouse. Notably, pituitary extract is a critical component of the *in vitro* 3D model of mammary morphogenesis [3, 4].

The extension of the model to other breast cancer cell lines as well as fresh breast cancer tissue was also demonstrated. In each case, the xenografts developed neo-vascularization, indicating that they had retained their angiogenic programming. Thus, the model has general utility for producing large amounts of tissue for further analysis.

In summary, the improved *in vivo* model allows the expansion of single-cell suspensions of mammary epithelial cells in a nascent murine mammary gland into a palpable xenograft within 4–8 weeks. This model uses fewer numbers of epithelial cells (0.2 million per xenograft) and allows them to establish, proliferate, and differentiate in the orthotopic site, which mimics mammary gland development and morphogenesis. The model requires Matrigel and MSCs for full engraftment, but does not require radiation-killed stromal cells, nor collagen I, thus preventing the undesirable overgrowth of stromal cells into fibrosarcomas. The improved model further allows the observation of xenograft growth in the presence or absence of pregnancy, thus providing additional insights into the effect of hormonal changes on xenograft development. Notably, our model was optimized for study of both mammary gland neoplasia and mammary gland morphogenesis. This model will allow us to evaluate the effect of adding in lymphocytes under inflammatory conditions to mimic the effect of chronic inflammation on human mammary cell differentiation or to test the effect of various treatments on tumor development. 

## Figures and Tables

**Table 1:** Requirement for preparation of mammary gland.

MEC ^1^	SC^2^	ECM	*N* ^3^	*x*/*T* ^4^	Outcome
*Humanized mammary fat pads*					
MCF7 (2 × 10^5^)	hTert MSC (2 × 10^5^)	Col I plus matrigel (3 : 1)	8	0/16	Solid tumor
MCF7/CEACAM1-4S (2 × 10^5^)	hTert MSC (2 × 10^5^)	Col I plus matrigel (3 : 1)	8	14/16*	Differentiation into striated epithelium

*Normal mouse mammary fat pads*					
MCF7 (2 × 10^5^)	hTert MSC (2X10^5^)	Col I plus matrigel (3 : 1)	8	0/16	Solid tumor
MCF7/CEACAM1-4S (2 × 10^5^)	hTert MSC (2X10^5^)	Col I plus matrigel (3 : 1)	8	14/15*	Differentiation into striated epithelium

^1^Mammary epithelial cell line (number of cells injected), ^2^Stromal cell line (number of cells injected), ^3^Number of animals, ^4^Number of xenografts exhibiting striated epithelial structure (*x*)/Total number of xenografts (*T*), *indicates statistical significance (*P* < .01) for differentiation of xenografts, when compared with control group (MCF7).

**Table 2:** Effect of extracellular matrix on differentiation of xenografts.

MEC ^1^	SC^2^	ECM	*N* ^3^	*x*/*T* ^4^	Outcome
None	hTert MSC (2 × 10^5^)	Matrigel orCol I plus matrigel (3 : 1)	8	—	No growth or no xenogratfs
MCF7 (2 × 10^5^)	None	Col I plus matrigel (3 : 1)	8	0/16	Solid tumor
MCF7/CEACAM1-4S (2 × 10^5^)	None	Col I plus matrigel (3 : 1)	8	0/16	Solid tumor and no differentiation
MCF7 (2 × 10^5^)	hTert MSC (2 × 10^5^)	None	8	0/4	Solid tumor and low engraftment
MCF7/CEACAM1-4S (2 × 10^5^)	hTert MSC (2 × 10^5^)	None	8	0/3	Solid tumor and low engraftment
MCF7 (2 × 10^5^)	None	Matrigel	8	0/16	Solid tumor
MCF7/CEACAM1-4S (2 × 10^5^)	None	Matrigel	8	15/16*	Differentiation into acini

^1^Mammary epithelial cellline (number of cells injected), ^2^Stromal cell line (number of cells injected), ^3^Number of animals, ^4^Number of xenografts exhibiting gland-like structures (*x*)/Total number of xenografts (*T*), *indicates statistical significance (*P* < .01) for differentiation of xenografts, when compared with all other groups in this table.

**Table 3:** Effect of type and number of stromal cells on differentiation of xenografts.

MEC ^1^	SC^2^	ECM	*N* ^3^	*x*/*T* ^4^	Outcome
MCF7 (2 × 10^5^)	hTert MSC (2 × 10^5^)	Matrigel	8	0/16	Solid tumor
MCF7/CEACAM1-4S (2 × 10^5^)	hTert MSC (2 × 10^5^)	Matrigel	8	15/16*	Differentiation into gland like structures
MCF7/CEACAM1-4S (2 × 10^5^)	hTert MSC (5 × 10^4^)	Matrigel	8	16/16*	Differentiation into gland like structures
MCF7/CEACAM1-4S (2 × 10^5^)	RMF/EG (2 × 10^5^)	Matrigel	8	0/16	Fibrosarcoma
MCF7 (2 × 10^5^)	RMF/EG (2 × 10^5^)	Matrigel	8	0/16	Fibrosarcoma

^1^Mammary epithelial cell line (number of cells injected), ^2^Stromal cell line (number of cells injected); ^3^Number of animals, ^4^Number of xenografts exhibiting gland-(acinar-) like structures (*x*)/Total number of xenografts (*T*), *indicates statistical significance (*P* < .01) for differentiation of xenografts, when compared with control group (MCF7).

**Table 4:** Characterization and optimization of an in vivo mammary gland morphogenesis model^1^.

MEC	Stromal cells	ECM	Outcome
*Humanized mammary fat pads*			
None	hTert MSC	ECM or None	No growth
+	hTert MSC	Col I plus matrigel	Slow growth of xenografts (>10 weeks) and differentiation into striated epithelium

*Normal mouse mammary fat pads*			
+	hTert MSC	Col I plus matrigel	Rapid xenograft growth (≤8 weeks) and differentiation into striated epithelium
+	hTert MSC	None	Low engraftment (3 out 8 animals) and lack of differentiation
+	None	Col I plus matrigel	Small xenografts (<1-2 mm) and lack of differentiation
+	None	Matrigel	Small xenografts (<1-2 mm), but formation of acni and clusters of cells
+	RMF/EG	Matrigel/No ECM	Larger xenografts (>5–10 mm), but outgrowth of fibroblasts
+	hTert MSC	Matrigel	Growth of xenografts (>5 mm) and differentiation epithelial cells

*Pregnant mammary fat pads*			
+	hTert MSC	Matrigel	Growth of xenografts (>5 mm) and formation of lumen

^1^+ indicates MCF-7 transfected with CEACAM1-4S.
