# Erratum of “Role of CEACAM1, ECM, and Mesenchymal Stem Cells in an Orthotopic Model of Human Breast Cancer”

**DOI:** 10.4061/2011/537380

**Published:** 2011-01-24

**Authors:** Sridhar Samineni, Carlotta Glackin, John E. Shively

**Affiliations:** ^1^Irell & Manella Graduate School of Biological Sciences, City of Hope, Duarte, CA 91010, USA; ^2^Department of Immunology, Beckman Research Institute, City of Hope, Duarte, CA 91010, USA; ^3^Department of Neurosciences, Beckman Research Institute, City of Hope, Duarte, CA 91010, USA

In the paper by Samineni et al. [1], the following corrections should be made: (1) page 3 column 2 line 18 should read “Figure  1(c),” (2) page 3 column 2 line 24 should read: “Figure  1(b),” (3) Figure  2 should be replaced with the attached new Figure 2. The figure legend remains the same.

## Figures and Tables

**Figure 2:**
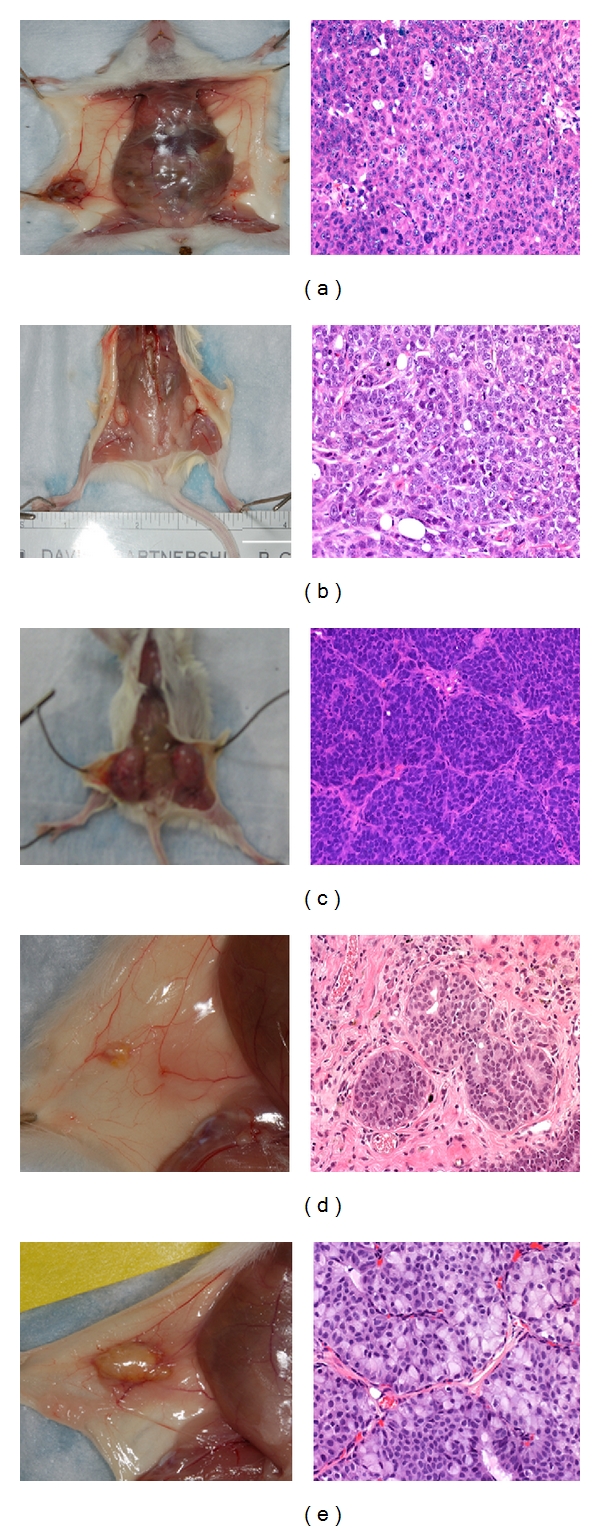
Growth and formation of xenografts by commonly used breast cancer cell lines or human breast tumor tissue in nascent mammary fat pad of NOD/SCID mice. Gross pictures of human xenografts (left panels) and corresponding H&E stained sections of xenografts (right panels) from breast cancer cell lines or human breast tissue implantation (a) SUM1315, (b) MDA-MB-468, (c) DU4475 (magnification 200x), (d) human breast epithelial organoids (magnification 400x), and (e) human breast tumor slices (magnification 200x).
